# Disparities in Utilization of Psychiatry Services Among Home Care Clients: The Tale of Two Canadian Jurisdictions

**DOI:** 10.3389/fpsyt.2021.712112

**Published:** 2021-09-17

**Authors:** Jeffrey Poss, Lori Mitchell, Jasmine Mah, Janice Keefe

**Affiliations:** ^1^School of Public Health and Health Systems, Faculty of Applied Health Sciences, University of Waterloo, Waterloo, ON, Canada; ^2^Winnipeg Regional Health Authority, Winnipeg, MB, Canada; ^3^Faculty of Medicine, Dalhousie University, Halifax, NS, Canada; ^4^Department of Family Studies and Gerontology, Mount Saint Vincent University, Halifax, NS, Canada; ^5^Nova Scotia Centre on Aging, Mount Saint Vincent University, Halifax, NS, Canada

**Keywords:** home care, older adults, mental health, physician visits, psychiatry services

## Abstract

Publicly funded home care in Canada supports older adults in the community to delay institutional care, which results in complex care populations with multimorbidity that includes mental health problems. The purpose of this study is to examine prevalence of psychiatric diagnoses and other mental health symptoms among older clients in two publicly funded Home Care (HC) Programs and their psychiatry service utilization (psychiatrist visits) after being admitted to home care. This retrospective cohort study examines clients age 60 years and older in the two Canadian provinces of Manitoba (MB), specifically the Winnipeg Regional Health Authority (WRHA) (*n* = 5,278), and Nova Scotia (NS) (*n* = 5,323). Clients were admitted between 2011 and 2013 and followed up to 4 years. Linked data sources include the InterRAI Resident Assessment Instrument for Home Care (RAI-HC), physician visit/billing data and hospital admission data. Both regions had similar proportions (53%) of home care clients with one or more psychiatric diagnoses. However, we observed over 10 times the volume of psychiatry visits in the WRHA cohort (8,246 visits vs. 792 visits in NS); this translated into a 4-fold increased likelihood of receiving psychiatry visits (17.2% of WRHA clients vs. 4.2% of NS clients) and 2.5 times more visits on average per client (9.1 avg. visits in MB vs. 3.6 avg. visits in NS). The location of psychiatry services varied, with a greater number of psychiatry visits occurring while in hospital for WRHA HC clients compared to more visits in the community for NS HC clients. Younger age, psychotropic medication use, depressive symptoms, dementia, and having an unstable health condition were significantly associated with receipt of psychiatry visits in both cohorts. Access to psychiatric care differed between the cohorts despite little to no difference in need. We conclude that many home care clients who could have benefitted from psychiatrist visits did not receive them. This is particularly true for rural areas of NS. By linking the RAI-HC with other health data, our study raises important questions about differential access to psychiatry services by site of care (hospital vs. community), by geographical location (MB vs. NS and urban vs. rural) and by age. This has implications for staff training and mental health resources in home care to properly support the mental health needs of clients in care. Study results suggest the need for a mental health strategy within public home care services.

## Introduction

Publicly-funded home care in Canada provides personal support and home health services to older adults in the community with the aim to keep clients safely living at home and to reduce hospital or long-term care facility admissions (1). Efforts to shift institutional care for older adults to community care have resulted in complex care populations in home care programs (2). Older adults using home care live with high levels of frailty and multimorbidity, including high rates of mental health problems (3).

There is a substantial body of literature on mental health in older adults. First, the high prevalence of mental health illnesses in this population is well-established. Among persons over the age of 65 years, the prevalence of mental health illnesses range from 17 to 30%, or as high as 40–50% if sub-clinical depression, anxiety, or dementia-related problems are included (4, 5). More than one-quarter of long-stay home care clients have one or more psychiatric diagnoses, which was shown to be associated with higher rates of home care and long-term care use (5, 6). Older adults therefore experience higher rates of mental health problems than the general Canadian population, where one in five Canadians live with a mental health illness each year (7).

Second, there are substantial and unique barriers faced by these older adults to diagnose and treat their illnesses. These intrinsic (i.e., attitudes of care providers, personal/cultural stigma and confounding medical comorbidities) and extrinsic (i.e., cost, transportation and reliance on caregivers) barriers are summarized in a recent systematic review by Lavingia et al. (8). Older adults living with mental health illness are often subjected to a double burden preventing referral to psychiatry services. While more comorbidities and loss of social connections are related to higher rates of depression, anxiety and other mental health problems, these same problems are often underreported by patients and under diagnosed by clinicians (4, 9). Referral to psychiatry services is impeded by the stigma surrounding mental health, the belief that mental illness is a normal part of the aging process and the complexity of distinguishing symptoms of mental illness from multifaceted medical conditions (8).

Lastly, there is good evidence to suggest older adults who receive diagnoses and treatment for psychiatric conditions have better health outcomes and quality of life than those who are not diagnosed (10). Even for certain diagnoses that are expected to progress or relapse over time, seeing a specialist can provide options to manage symptoms, promote recovery and improve well-being for older adults and their caregivers (10). The Mental Health Commission of Canada identifies four unique populations of older adults who benefit from specialists with additional psychiatric training: (i) older adults with recurrent, persistent or chronic mental illness; (ii) older adults with late onset mental illnesses; (iii) older adults with behavioral and psychological symptoms associated with dementias, and (iv) older adults with chronic medical problems with known correlations with mental illnesses (e.g., cerebral vascular disease) (10). Additionally, substance misuse and suicidal ideation are highly prevalent in this population (10). Understanding and managing these unique presentations of mental health takes training and specialized knowledge.

What has not been systematically documented or explored is the magnitude of difference between need of psychiatry services and receipt of these services. Even in a country with universal health care such as Canada, it is generally purported that mental health services have been neglected (7, 11). Underfunding, narrow public insurance coverage, geography (urban vs. rural, provincial variation) and unavailability of primary care services have contributed to high rates of unmet needs and inaccessibility of specialized services (11–13). Of Canadians needing mental health care in 2018, 41.2–60.3% self-reported having unmet or only partially met needs, with particular deficits in finding counseling or therapy for psychiatric issues (13). Furthermore, there is even less evidence to compare unmet needs in older adults with mental health diagnoses, gaps which are hypothesized to be even greater than the general population. In one American study, only 5% of users of publicly funded mental health services were older adults despite comprising 20% of the study population. Other studies have shown a reluctance of older adults compared with younger adults to use psychiatry services after a mental health diagnosis (14, 15). Availability of specialized services can be particularly challenging for older adults with psychiatric diagnoses who live in rural areas. Overall, there are fewer psychiatrists and mental health services in rural areas of Canada, where healthcare resources including psychiatry services are clustered in the larger urban areas (16, 17). In rural areas, which represent the highest proportion of older people in their populations, responsibility for delivery of psychiatric care often falls to other services (e.g., emergency departments) in the absence of dedicated mental health facilities (18). To our knowledge, there is no evidence comparing need for mental health services and prevalence of seeing a psychiatrist in older adults in different areas in Canada. This research can help us better understand geographical and age-related differences in access to mental health care and guide areas for potential interventions to improve care for this population.

As the Canadian senior population grows, combined with the increased shift of older adult care to the community, home care services will increasingly be expected to help older adults with mental health conditions. Home care will need to collaborate with specialized mental health services to best care for this increasingly multi-morbid population. This study examines the prevalence of psychiatric diagnoses and other mental health symptoms among older clients in two publicly funded Home Care Programs in Canada: the province of Nova Scotia (NS) and the Winnipeg Regional Health Authority (WRHA) in the province of Manitoba (MB). In addition, we examine the psychiatry service utilization (psychiatrist visits) by home care clients.

## Materials and Methods

### Study Setting and Population

This study is part of a larger research program examining the pathways of older adults with chronic and long term conditions through home care, in the large urban centre serviced by the WRHA and the province of Nova Scotia as a whole (19). The study population included older adults, age 60 or older at time of admission to home care, who were long-stay clients (receiving service for 60 days or longer) in the publicly funded Home Care Programs in the WRHA or Nova Scotia. Including all of Nova Scotia in the study provided the opportunity to examine both urban and rural perspectives.

In both jurisdictions, a range of mental health services are provided in the community and in hospital. Home care clients usually have to be seen by another healthcare professional who makes a referral to psychiatry services, usually in the form of an outpatient clinic visit. Referrals can be made through a family physician, a nurse, an emergency physician, or allied health professionals who work with Home Care. Referral by a family physician or home care staff can be made to Community Mental Health Services for community follow up. Both settings have mental health and addictions crisis lines. Both provinces list directories of government, health and community resources online or through a telephone number. Telephone self-referrals, through a Central Intake service were available in the WRHA during the period of this study (20). In Nova Scotia, some in-patient mental health services operated only in the largest urban centre in the province, the city of Halifax. Notably, the only in-patient program for geriatric psychiatry is located in Halifax (21).

Older adults with mental illnesses may also be referred to a geriatrician or geriatric psychiatrist. Geriatric outreach teams are a cornerstone of specialized services for older adults in both provinces (4, 10), consisting of multidisciplinary team members who take a holistic approach to assessment, consultation, treatment and education for clients and their families with the goal of helping clients remain at home (4). These outreach teams, as well as geriatric day programs, often have access to specialized mental health professionals, illustrating another pathway by which home care clients can access psychiatry services.

### Study Design and Data Sources

A retrospective cohort study was conducted to examine the mental health of home care clients and their use of psychiatry services. Several clinical and administrative data sources were utilized. Data sources from both WRHA and Nova Scotia jurisdictions were substantially the same, and included:

- Resident Assessment Instrument for Home Care (RAI-HC) (22) is mandated in both jurisdictions as part of regular clinical practice. The RAI-HC is a standardized, comprehensive assessment for use with adult and non-palliative home care clients. It is the data standard for the Canadian Institute for Health Information (CIHI) national reporting system for home care (23), and has acceptable reliability and validity (24, 25). Adult home care clients in both jurisdictions, expected to be on service at least 60 days, receive an initial assessment on referral to home care, and are expected to be re-assessed annually, or earlier in the case of a significant clinical change. The software for electronic completion of the assessment in both jurisdictions ensures that assessments are fully completed, thereby providing RAI-HC data without any missing values.
- Discharge Abstract Database (DAD) is the CIHI standard for acute care hospitalizations and is used here to inform mental health ICD-10 diagnoses assigned during a hospitalization within the home care episode.
- Vital statistics were provided to inform dates of death, as part of understanding discharge from home care.
- Physician administration records, either used for direct billing or as part of shadow-billing (where non-fee-for-service payment is in place) during the home care episode. Data included the physician specialty, date of service, location of service, and an ICD-9 diagnosis code.

Two cohorts were selected in an equivalent manner for each jurisdiction, the criteria being that a home care client age 60 or older received an initial RAI-HC assessment between January 1, 2011 and December 31, 2013, and at least one additional RAI-HC assessment in the following 4 years. They also had to have been active on home care for at least 60 days, and received some home support service (e.g., assistance with tasks such as bathing or dressing) in the first 120 days of their home care episode. The criterion that most greatly restricted cohort selection (~30% of potential cases in NS and 20% in the WRHA) was the home support service requirement. Those selected, at the time of their initial assessment, were more likely to have had a recent decline in physical independence, not have a diagnosis of dementia or significant cognitive impairment, and have experienced a recent stay in hospital. Those not selected were more likely to have refused service or be deemed ineligible. The review period for this cohort was from the time of the initial assessment until discharge from home care, up to 4 years from the initial assessment.

For the analyses, the WRHA was treated as a single geographic area, containing no rural areas. Nova Scotia was further sub-divided into three zones based on the home care client's postal forward sortation area (FSA) at their initial assessment:

- Halifax and near-vicinity, based on FSA that mapped to Halifax or to an area with a strong or moderate metropolitan influence (26) of Halifax;
- Rural areas, denoted by a zero as the second digit of the FSA (27);
- Non-Halifax urban, all others.

Mental health diagnoses were assigned from a chosen list of ICD-9 and ICD-10 codes, listed in the Supplementary Material. Note that this comprised a broad range of mental health conditions but excluded those for Alzheimer's disease or a related dementia. Dementias were excluded as these are neurocognitive disorders and not mental illnesses *per se*. Excluding dementias from the list of diagnoses provided a better focus on mental illness in home care.

Client characteristics were drawn from the RAI-Home Care assessment items, and included demographic items, current psychotropic medications, a scale of depressive symptoms i.e., Depression Rating Scale (DRS) (28) and one for risk of long-term care facility placement i.e., Method for Assigning Priority Levels (MAPLe) (29), and other symptoms of anxiety or psychosis. Discharge status up to the 4-year period of follow-up was also assigned.

Physician visits with the specialty of psychiatry (i.e., psychiatry visits) from the physician administration records were identified that occurred within that client's home care episode.

Statistical testing across groups used chi-square tests for dichotomous variables, and *t*-tests for continuous variables.

Sensitivity analyses were conducted to shed light on how those in the cohorts with dementia who were more likely to exhibit neuropsychiatric symptoms differed in their likelihood of being seen by psychiatrist, or receiving psychotropic medications.

A multivariable logistic regression model was conducted, for each jurisdiction, on the likelihood of the home care client receiving one or more psychiatry visits, utilizing covariates used in the descriptive analysis. All variables were retained in the model, regardless of their significance.

Ethical approval for this study was obtained from Nova Scotia Health, Mount Saint Vincent University and the University of Manitoba. Research approval and data access was obtained from Manitoba Health, Seniors and Active Living's Health Information Privacy Committee (HIPC) and the WRHA's Research Access and Approval Committee. HIPC provided approval to access the physician administration data for the WRHA cohort from the National Physician Database at the Canadian Institute for Health Information.

All analyses were conducted with SAS v 9.4.

## Results

There were 5,278 cases in the WRHA cohort, and 5,323 in NS. Within the NS cohort, 1,306 (24.5%) were in the Halifax area, 1,755 (33.0%) were in urban areas outside of Halifax, and 2,262 (42.5%) were rural (Table 1).

**Table 1:** Psychiatry services, by province, and urban/rural.

	**WRHA**	**Nova Scotia**			
		**All**	**Halifax**	**Urban non-Halifax**	**Rural**
Home care clients in study (*N*)	5,278	5,323	1,306	1,755	2,262
#Clients with psychiatry service (%)	908 (17.2)	223 (4.2)	93 (7.1)	84 (4.8)	46 (2.0)
#Of psychiatry visits	8,246	793	338	330	125
Avg. #of visits among clients with psychiatry	9.1	3.6	3.6	3.9	2.7
#Of unique psychiatrists	107	58	35	17	14
#Of psychiatry visits provided in hospital (%)	6,478 (78.6)	222 (28.0)	143 (42.3)	45 (13.6)	34 (27.2)
#Of psychiatry visits provided in community (%)	1,768 (21.4)	571 (72.0)	195 (57.7)	285 (86.4)	91 (72.8)

Table 1 summarizes the psychiatry visit data for the two cohorts, with further stratification by location of the NS cohort. Across all years of service data following these cohorts (2011–2017) there were 107 different psychiatrists in the WRHA that provided 8,246 visits, and 58 different psychiatrists in NS that provided 793 visits. In the WRHA 79% of the visits were reported to have occurred in hospital, compared to 26% of those in NS.

In the WRHA, 908 (17.2%) received one or more visits by a psychiatrist during their home care episode, compared to 223 (4.2%) in the NS cohort. WRHA psychiatry recipients averaged 9.1 psychiatry visits, while those in NS averaged 3.6 visits.

Table 2 presents selected characteristics by provincial cohort, stratified by receipt of any psychiatry service. Consistently significant differences in both cohorts were found. Those receiving psychiatry services were younger in age, more likely to be receiving psychotropic medications, have a diagnosis of Alzheimer's disease or a related dementia, exhibit depressive symptoms, have a condition considered to be unstable, be at high risk for long-term care admission, not be at ease with others, and have hallucinations. In addition, within the WRHA cohort, clients receiving psychiatry services were more likely to not have an informal caregiver, to have made recent economic trade-off decisions because of limited funds, to have delusions, and to use tobacco daily. Discharge disposition groups differed significantly among the WRHA cohort only, with the clients who received psychiatry more likely to enter a long-term care facility.

**Table 2:** Sample characteristics at latest RAI-HC assessment, those with and without psychiatry visit.

**(%Of the column, unless indicated otherwise)**	**WRHA**			**Nova Scotia**			*p* value WRHA vs. NS^*^
**Received any psychiatry while on home care**	**No**	**Yes**	***p***	**No**	**Yes**	***p***	
*N*	4,370	908		5,100	223		
(% of provincial cohort)	82.8	17.2		95.8	4.2		
Mean age in years (standard deviation)	84.3 (8.1)	80.6 (8.7)	**<0.0001**	83.1 (8.3)	79.2 (8.1)	**<0.0001**	**0.029**
Female	66.8	63.2	**0.036**	69.7	70.0	0.922	0.059
Married	31.8	34.8	0.077	30.2	33.2	0.345	0.207
No informal caregiver	1.1	2.2	**0.011**	1.7	3.1	0.093	0.412
Made economic trade-offs due to limited funds	2.8	4.9	**0.001**	4.0	4.0	0.955	0.608
Antipsychotics in the past 7 days	5.6	24.5	**<0.0001**	10.6	24.7	**<0.0001**	0.947
Anxiolytics in the past 7 days	9.1	21.2	**<0.0001**	17.7	31.4	**<0.0001**	**0.001**
Antidepressants in the past 7 days	16.3	39.5	**<0.0001**	33.1	54.3	**<0.0001**	**<0.0001**
Alzheimer's/related dementia	26.5	42.6	**<0.0001**	34.9	48.4	**<0.0001**	0.117
Depressive symptoms (DRS 3+)	8.9	23.7	**<0.0001**	26.4	42.2	**<0.0001**	**<0.0001**
Condition makes cognition, mood, ADL or behavior unstable	42.8	62.8	**<0.0001**	48.7	69.1	**<0.0001**	0.080
High risk of long-term care home entry (MAPLe 4 or 5)	40.8	56.0	**<0.0001**	54.6	67.7	**<0.0001**	**0.001**
Not at ease with others	4.8	11.8	**<0.0001**	8.8	13.0	**0.029**	0.616
Hallucinations	1.2	4.2	**<0.0001**	3.4	5.8	**0.050**	0.289
Delusions	1.5	4.6	**<0.0001**	5.4	7.2	0.252	0.122
Tobacco use daily	5.5	8.6	**0.001**	8.8	11.7	0.146	0.155
Discharge Disposition from Home Care			**<0.0001**			0.222	0.318
• Discharged deceased	19.6	14.8		18.4	16.6		
• Discharged to long-term care facility	30.1	43.7		43.1	49.3		
• Remain on home care after 4 years	39.4	32.3		30.0	28.3		

* *Comparing those in the WRHA receiving any psychiatry with those in Nova Scotia receiving any psychiatry*.

*DRS, Depression Rating Scale, sum of 7 depressive symptom items, range 0 to 14, higher scores are more severe; ADL, Activities of daily living; MAPLe, Method for Assigning Priority Level, algorithm assigning 1–5 range, with higher scores more at risk of long-term care placement and caregiver distress. Bolding denotes p values significant at the 0.05 (95% confidence) level*.

Table 2 also summarizes differences *between* the two provincial cohorts among those who received psychiatry services. Here there are fewer differences found, although NS cases receiving psychiatry services were younger, more likely to receive anxiolytic or antidepressant medications, have more depressive symptoms, and were at greater risk of long-term care placement, compared to WRHA cases receiving psychiatry services.

Table 3 presents the same characteristics as Table 1, but stratified based on the presence of any mental health diagnosis while on home care. Remarkably, the prevalence of such a diagnosis did not differ across the cohorts, at 53%. Similar to receipt of psychiatry services, those with a diagnosis were consistently more likely in both cohorts to be younger, to be receiving psychotropic medications, have a diagnosis of Alzheimer's disease or related dementia, have depressive symptoms, an unstable condition, be at high risk of long-term care placement, be not at ease with others, exhibit hallucinations, and have daily tobacco use. In addition, those in NS with a mental health diagnosis were more likely to have made recent economic trade-off decisions due to limited funds, while those in the WRHA had significantly higher prevalence of delusions. In both cohorts, those with a mental health diagnosis were more likely to be discharged from home care to a long-term care facility.

**Table 3:** Sample characteristics at latest RAI-HC assessment, those with and without mental health diagnosis.

**(%Of the column, unless indicated otherwise)**	**WRHA**			**Nova Scotia**			p value WRHA vs NS^*^
Any mental health diagnosis while on home care^**^	**No**	**Yes**	***p***	**No**	**Yes**	***p***	
*N*	2,483	2,795		2,495	2,828		
(% of provincial cohort)	47.0	53.0		46.9	53.1		
Mean age in years (standard deviation)	84.6 (8.2)	82.8 (8.4)	**<0.0001**	84.2 (8.2)	81.8 (8.3)	**<0.0001**	**<0.0001**
Female	65.2	67.1	0.142	68.7	70.5	0.151	0.340
Married	31.5	33.0	0.260	29.1	31.4	0.073	0.638
No informal caregiver	1.1	1.5	0.153	1.4	1.9	0.159	0.440
Made economic trade-offs due to limited funds	2.7	3.5	0.092	3.3	4.5	**0.025**	0.497
Antipsychotics in the past 7 days	3.3	13.7	**<0.0001**	6.5	15.4	**<0.0001**	0.539
Anxiolytics in the past 7 days	4.6	17.0	**<0.0001**	9.0	26.5	**<0.0001**	**0.001**
Antidepressants in the past 7 days	7.7	31.4	**<0.0001**	19.8	46.5	**<0.0001**	**<0.0001**
Alzheimer's/related dementia	21.8	36.0	**<0.0001**	29.3	41.0	**<0.0001**	0.184
Depressive symptoms (DRS 3+)	6.8	15.5	**<0.0001**	20.1	33.2	**<0.0001**	**<0.0001**
Condition makes cognition, mood, ADL or behavior unstable	37.4	54.2	**<0.0001**	41.9	56.2	**<0.0001**	0.616
High risk of long-term care home entry (MAPLe 4 or 5)	36.8	49.3	**<0.0001**	50.6	59.1	**<0.0001**	**0.008**
Not at ease with others	4.2	7.7	**<0.0001**	6.5	11.1	**<0.0001**	0.091
Hallucinations	0.8	2.5	**<0.0001**	2.4	4.4	**<0.0001**	0.121
Delusions	1.2	2.7	**<0.0001**	5.0	5.9	0.134	**0.022**
Tobacco use daily	4.2	7.8	**<0.0001**	6.7	10.9	**<0.0001**	0.156
Discharge Disposition from Home Care			**<0.0001**			**<0.0001**	0.131
• Discharged deceased	22.0	15.8		20.9	16.1		
• Discharged to long-term care facility	25.7	38.4		40.7	45.6		
• Remain on home care after 4 years	40.7	35.9		29.9	30.0		

* *Comparing those in the WRHA receiving any psychiatry with those in Nova Scotia receiving any psychiatry*.

** *Any of: psychiatric condition recorded on any RAI-HC assessment, selected ICD-9 diagnoses from physician visits while home care clients, selected ICD-10 diagnoses from hospital admissions while home care clients, excludes dementia and delirium*.

*DRS, Depression Rating Scale, sum of 7 depressive symptom items, range 0 to 14, higher scores are more severe*.

*ADL, Activities of Daily Living*.

*MAPLe, Method for Assigning Priority Level, algorithm assigning 1–5 range, with higher scores more at risk of long-term care placement and caregiver distress. Bolding denotes p values significant at the 0.05 (95% confidence) level*.

Further, some differences between the provincial cohorts with a mental health diagnosis were evident: NS clients were younger, more likely to be taking an anxiolytic or antidepressant medication, have more depressive symptoms, be at higher risk of long-term care placement, and exhibit delusions.

Table 4 provides additional details regarding psychiatric diagnoses, including six mental health diagnoses, stratified by cohort and further in NS by Halifax/urban non-Halifax/rural. These groups were limited by the truncated values of ICD-9 codes, with some common groups like mood disorders impossible to aggregate. In NS, clients in or near Halifax were more likely to have seen a psychiatrist than in other urban areas. Those in rural NS areas were less likely than those in urban areas outside of Halifax, despite there being no difference overall in the prevalence of a mental health diagnosis.

**Table 4:** Characteristics of HC clients having any psychiatry and/or mental health diagnoses, and by selected diagnostic groups, by province and urban/rural.

	**WRHA**	**Nova Scotia**				***p***		
		**All**	**Halifax**	**Urban**	**Rural**	**WRHA vs. NS**	**Halifax vs**.	**Urban non-Halifax**
				**non-Halifax**			**urban non-Halifax**	**vs. Rural**
Home care clients (*N*)	5,278	5,323	1,306	1,755	2,262			
Any psychiatry (%)	17.2	4.2	7.1	4.8	2.0	**<0.0001**	**0.012**	<0.0**001**
Any mental health diagnosis (%)	53.0	53.1	52.3	55.1	52.1	0.905	0.124	0.061
Any psychiatry among those with MH diagnosis (%)	27.4	7.4	13.2	8.1	3.6	**<0.0001**	**0.001**	**<0.0001**
Any psychiatry among those without MH diagnosis (%)	5.7	0.5	0.5	0.8	0.4	**<0.0001**	0.512	0.250
**Anxiety disorder diagnosis**								
Prevalence (%)	23.1	20.3	20.7	20.3	20.1	**0.0005**	0.792	**0.018**
Any psychiatry among those (%)	29.1	10.0	17.0	11.2	4.8	**<0.0001**	**0.037**	**0.001**
**Psychoses diagnosis**								
Prevalence (%)	13.4	15.8	19.6	17.9	12.0	**0.0005**	0.229	**<0.0001**
Any psychiatry among those (%)	49.4	12.0	21.5	10.8	4.4	**<0.0001**	**0.0005**	**0.004**
**Acute reaction to stress or adjustment disorder Diagnosis**								
Prevalence (%)	4.4	4.3	6.0	3.9	3.5	0.813	**0.007**	0.522
Any psychiatry among those (%)	41.4	21.2	33.3	18.8	11.3	**<0.0001**	0.053	0.191
**Diagnosis related to special symptoms including sleep, pain disorder**								
Prevalence (%)	1.9	5.6	6.1	6.1	5.0	**<0.0001**	0.974	0.128
Any psychiatry among those (%)	24.2	10.7	19.0	^*^	^*^	**0.001**	0.211	**0.017**
**Diagnosis related to alcohol or drugs**								
Prevalence (%)	1.0	2.6	2.6	2.4	2.8	**<0.0001**	0.712	0.440
Any psychiatry among those (%)	50.0	7.2	14.7	^*^	^*^	**<0.0001**	0.487	0.061
**Personality disorder diagnosis**								
Prevalence (%)	0.7	0.6	0.6	0.8	0.5	0.523	0.549	0.213
Any psychiatry among those (%)	62.9	29.4	^*^	35.7	^*^	**0.005**	0.512	0.122

* *Cell suppressed due to fewer than 5 persons. These are p values significant at the 0.05 (95% confidence) level*.

In both sites, clients with a mental health diagnosis had a significantly higher rate of psychiatry visits than clients without a mental health diagnosis (Table 4). In the WRHA, 27.4% of clients with a mental health diagnosis had psychiatry services compared to only 5.7% of clients without a mental health diagnosis (nearly 5 times the rate). Similarly, in NS 7.4% of clients with a mental health diagnosis had psychiatry services compared to only 0.5% of clients without a mental health diagnosis (over 14 times the rate). However, in NS, among those with a mental health diagnosis, the likelihood of having received a psychiatry visit decreased significantly going from Halifax (13.2%) to urban non-Halifax (8.1%), to rural (3.6%). Among those without a mental health diagnosis there is no such pattern with rates below 1%.

Anxiety disorder was present in more than 20% of both cohorts, and more prevalent in the WRHA cases. Psychosis was diagnosed in at least 13% of cases, with prevalence higher in NS. Likelihood of receiving psychiatry service was consistently higher in the WRHA cohort regardless of diagnosis group. Patterns of lower likelihood of psychiatry service outside of Halifax were also evident among most of the selected diagnoses, except for some rarer groups where there is limited statistical power.

We investigated total days of care that were observable between and within the WRHA and NS cohorts, and there were no significant sources of bias that would result from differential observation periods as home care clients.

A sensitivity analysis applied 3-level stratification to the cohorts: those with a dementia diagnosis, with no dementia but with a psychiatric diagnosis, and others, as shown in Appendix A. All selected measures show significant differences, within cohorts, except for antipsychotics in NS with similar proportions comparing dementia and non-dementia with psychiatric diagnosis. Psychotic symptoms of hallucinations and delusions had the highest prevalence among those with dementia in both cohorts. Psychotropic medications are most often prescribed, and psychiatry is most often received by those with a psychiatric diagnosis where dementia is absent, followed by those with dementia, and those with no dementia and no psychiatric diagnosis. Those with dementia in the WRHA and NS cohorts had a similar prevalence of comorbid psychiatric diagnosis (21%).

Results of the multivariable logistic models on the likelihood of the receipt of one or more psychiatry visits are provided as Table 5. Significance and direction of odds ratios may be compared, but the magnitudes of odds ratios are not comparable due to the large difference in the prevalence of the dependent variable between provincial cohorts. For five covariates that are significant in both models (three psychotropic medications, depressive symptoms, and unstable condition), the direction of the effect is consistent between the jurisdictions. In the WRHA sample, older age was protective and not being at ease with others was protective against psychiatry visits, while a dementia diagnosis, high MAPLe scores, and hallucinations were predictive. Model fit was stronger in the WRHA data.

**Table 5:** Multivariable logistic regression models of clients receiving any psychiatry visits within each provincial cohort.

	**WRHA (** ***n*** **= 5,278 and 908**			**Nova Scotia (** ***n*** **= 5,323 and 223**		
	**received psychiatry visit)**			**received psychiatry visit)**		
	**Wald chi-square**	***P***	Odds ratio^*^	**Wald chi-square**	***P***	Odds ratio^*^
**Age group: 60–64**	**Ref**.	**Ref**.	**Ref**.	**Ref**.	**Ref**.	**Ref**.
65–74	0.13	0.718	0.92	0.22	0.638	1.26
75– 84	5.02	0.025	**0.60**	0.21	0.648	0.80
85 and older	16.31	<0.0001	**0.40**	2.04	0.153	0.49
Female	3.57	0.059	0.85	0.34	0.560	1.10
Married	0.16	0.689	0.96	1.20	0.273	0.84
No informal caregiver	2.02	0.156	1.56	3.43	0.064	2.18
Made economic trade-offs due to limited funds	0.47	0.493	1.15	1.07	0.299	0.69
Antipsychotics in the past 7 days	110.94	<0.0001	**3.33**	11.88	0.001	**1.82**
Anxiolytics in the past 7 days	32.10	<0.0001	**1.79**	10.92	0.001	**1.68**
Antidepressants in the past 7 days	37.53	<0.0001	**1.96**	16.89	<0.0001	**1.80**
Alzheimer's/related dementia	110.26	<0.0001	**2.50**	2.43	0.119	1.31
Depressive symptoms (DRS 3+)	47.42	<0.0001	**2.10**	4.86	0.028	**1.40**
Condition makes cognition, mood, ADL or behavior unstable	13.74	0.000	**1.38**	8.40	0.004	**1.64**
High risk of long-term care home entry (MAPLe 4 or 5)	11.89	0.001	**1.40**	1.84	0.174	1.27
Not at ease with others	17.88	<0.0001	**0.59**	0.05	0.827	0.95
Hallucinations	6.41	0.011	**1.44**	0.02	0.888	1.05
Delusions	1.56	0.211	1.38	0.39	0.532	0.83
Tobacco use daily	2.74	0.098	1.50	0.05	0.823	1.05
C-Statistic (area under ROC Curve)	0.761			0.725		

* *Caution to not directly compare odds ratios between WRHA and Nova Scotia, since the rate of receipt of psychiatry visits differs between the two jurisdictions. These are p values significant at the 0.05 (95% confidence) level*.

## Discussion

This study provides important new information about mental health and illness among older home care clients and their receipt of psychiatry services. Information drawn from the RAI-Home Care along with administrative data including physician visits provide a powerful means for comparing and contrasting individuals receiving services in these two Canadian jurisdictions. Prevalence of mental health diagnoses was found to be equally high in both home care study cohorts, higher than found in previous reviews (6, 7). Yet overall, we observed over 10 times the volume of psychiatry visits in the WRHA cohort (8,246 visits vs. 792 visits in NS) which translated into a 4-fold increased likelihood of receipt of any psychiatry visits (17.2% of WRHA clients vs. 4.2% of NS clients), and 2.5 times more visits on average per client (9.1 avg. visits vs. 3.6 avg. visits in NS) among those home care recipients.

Client characteristics from the RAI-Home Care differed in ways that paints the NS cohort as having somewhat higher and more complex needs: higher prevalence of dementia, depressive symptoms, hallucinations, and delusions. The higher proportion of the NS cohort with elevated MAPLe scores is notable, since it brings together multiple factors related to caregiver burnout and risk of long-term care placement, including physical and cognitive impairment, history of falls, and responsive dementia behaviors (29).

As expected, patterns are similar whether the comparison is of those receiving psychiatry services, or those with a mental health diagnosis, since there is a strong relationship between any mental diagnosis and receiving a psychiatry visit. A client is nearly 5 times more likely to have a psychiatry visit in the WRHA when a mental health diagnosis is evident; and over 14 times more likely in NS. Similar to previous literature, this study found prevalence of psychiatry services was higher in urban than in rural settings (12, 14). In fact, psychiatry services were almost non-existent for rural Nova Scotians and leads to questions as to a higher burden on family physicians in these areas.

Both settings in this study have similar rates of psychiatrists available for their respective populations. According to the 2019 Canadian Medical Association Psychiatry Profile, the number of psychiatrists was 13.3 per 100,000 population in Manitoba and 14.5 per 100,000 population in Nova Scotia (30). In contrast, NS has 1.1 geriatricians per 100,000 persons and Manitoba has 0.4 per 100,000 persons. Some of the differences in psychiatry visits in our two populations may be due to geriatrician availability for specialty services among older home care clients with mental health issues in Nova Scotia. Future research is required to explore this relationship further. Research is also needed to assess the effect age has on the use of psychiatry services among home care clients. Nova Scotia has both a higher prevalence (21.1%) and higher numbers of older persons in its population (208,825 in 2020) (31) compared to WRHA (16.2 %; 127,032 in 2019) (32), yet cohort sizes were similar. We found, among other characteristics, younger home care users were more likely to use psychiatry services. With the higher level of needs and complexities among NS home care clients, one wonders whether referrals for scarce psychiatry services also suffer from age discrimination, stigma of mental health services or assumptions that mental illness is a normal part of the aging process. These are only speculations—but warrant further investigation.

A much larger proportion of the WRHA cohort accessed psychiatry services while hospitalized rather than in a community setting, while the opposite was found for the Nova Scotia cohort. An in-depth review of community mental health services and psychiatry services available in both sites was beyond the scope of the present study. Such a review may aid in identifying if there is differential focus on the care setting for mental health needs, and which mental health professionals are involved in the care. Previous systematic reviews found community mental health teams can have an impact on hospital admissions and lengths of stay. Similarly in-patient mental health bed supply can affect the amount of psychiatry services provided in hospital (33, 34).

The characteristics of the clients in both settings identified a high prevalence of dementia, ranging from 26.5% (WRHA) to 34.9% (Nova Scotia). For the purpose of this study a dementia diagnosis was not included in the mental health diagnostic areas of concern. As a cognitive disorder, individuals with Alzheimer's and related dementias are often referred to neurologists, geriatricians or serviced by primary care practitioners. However, it is recognized that there is a relationship between dementia and psychiatric disorders (35). Some of the psychiatry service results found in this study could be influenced by dementia diagnoses as opposed to other mental health issues, or if present, the comorbidity of mental illness and dementia.

This descriptive study is strengthened by population level data, high quality administrative data, and the availability of comparable measures and data in the different jurisdictions. Results identified that psychiatry visits represent a smaller proportion of medical service visits for older home care clients. Due to the study approach employed, the results are limited to their descriptive nature, that is, the observation of the very large difference in utilization of this specialized health service provider between two Canadian jurisdictions. However, the results cannot directly report on effective mental health services or outcomes.

In addition, the study is limited by looking at home care client characteristics at one point in time while reviewing receipt of psychiatry services over a time span up to 4-years in length. The characterization of the home care client may not be reflective of client status when visiting a psychiatrist, and the single point-in-time RAI-HC assessment results in symptom prevalence values that are under-estimates of what would be observed across the entire episode of home care. However, by using the client's most recent available assessment, it is more likely to capture any deterioration or changes in clinical status that may have prompted use of psychiatry services throughout the episode of home care.

## Conclusion

In the current study, the prevalence of mental health diagnoses was high among older adult home care clients, and higher than found in the general population in Canada. Despite the higher prevalence, visits to a psychiatrist were low by comparison. The results from this study suggest the need for a mental health strategy within public home care services. Publicly-funded home care in Canada sits largely outside of the medical model, and psychiatry visits represent a small proportion of medical services visits for these clients—representing a thin edge when considering the overall picture. Nevertheless, our descriptive study has raised important questions about differential access to psychiatry services by site of care (hospital vs. community), by geographical location (MB vs. NS and urban vs. rural) and by age. We acknowledge our data only provide a snapshot and call for more research in this area.

## Data Availability Statement

The data analyzed in this study is subject to the following licenses/restrictions: This study was conducted with data held by regional health and provincial authorities and the national body of the Canadian Institute for Health Information (CIHI). None of the data utilized are in the public domain. Access to the data is restricted to members of the research team based on strict data access and sharing agreements. From our Agreements with our Health administrative data sources, the following restrictions are applied to Publication of Data: The Applicant retains the right to publish research findings provided that results reported include only summary data and statistical analysis that preclude the identification, either alone or with other information, of subject individuals. For further information to access any data held by: WRHA, contact the WRHA Research Access and Approval Committee; Manitoba Health, contact the Manitoba Health, Seniors and Active Living Health Information Privacy Committee; hipc@gov.mb.ca; Health Data Nova Scotia (HDNS), contact HDNS@dal.ca; CIHI: https://www.cihi.ca/en/access-data-and-reports/make-a-data-request.

## Ethics Statement

The studies involving human participants were reviewed and approved by Nova Scotia Health Research Ethics Board-Reference NSHA REB ROMEO FILE #: 1024323; Mount Saint Vincent Research Ethics Board Certificate of Research Clearances for NS-File #: 2018–202; Mount Saint Vincent Research Ethics Board Certificate of Research Clearances for MB-File #: 2018-087; University of Manitoba Health Research Ethics Board Certificate of Final Approval for New Studies Ethics #: HS22118 (H2018:349. The ethics committee waived the requirement of written informed consent for participation.

## Author Contributions

JP contributed to the study design, analysis and interpretation of the data, and drafting of the manuscript. LM contributed to the study design, interpretation of the data, and drafting of the manuscript. JM and JK contributed to interpretation of the data and drafting of the manuscript. JK is the Principal Applicant for the CIHR operational grant that funded this work. All authors were involved in critical revisions to the manuscript and approve of the submitted version to be published.

## Funding

This study was funded by the Canadian Institutes of Health Research (CIHR).

## Author Disclaimer

The data used in this report were made available by Health Data Nova Scotia of Dalhousie University, the Winnipeg Regional Health Authority and the Canadian Institute for Health Information (with permission from Manitoba Health, Seniors and Active Living). Although this research is based on data obtained from the Nova Scotia Department of Health and Wellness, the Winnipeg Regional Health Authority, and the Canadian Institute for Health Information. The analyses, observations, conclusions, and opinions expressed are those of the authors and do not represent those of either Health Data Nova Scotia or the Department of Health and Wellness, Manitoba Health, Seniors and Active Living, the Winnipeg Regional Health Authority or the Canadian Institute for Health Information. We also thank the Geriatric Psychiatry Programs at the University of Manitoba and Dalhousie University for their contributions to the background of our paper.

## Conflict of Interest

The authors declare that the research was conducted in the absence of any commercial or financial relationships that could be construed as a potential conflict of interest.

## Publisher's Note

All claims expressed in this article are solely those of the authors and do not necessarily represent those of their affiliated organizations, or those of the publisher, the editors and the reviewers. Any product that may be evaluated in this article, or claim that may be made by its manufacturer, is not guaranteed or endorsed by the publisher.

## Appendix

**Appendix A:** Proportion of HC Clients with specific mental health characteristics by dementia diagnosis, and province.

	**WRHA**			**Nova Scotia**		
	**Dementia**	**No dementia**,	**No dementia**,	**Dementia**	**No dementia**,	**No dementia**,
		**psych dx**	**no psych dx**		**psych dx**	**no psych dx**
*n*	1,654	682	2,942	1,888	744	2,691
Hallucinations	5.3%	1.9%	0.5%	11.1%	3.4%	2.1%
Delusions	4.2%	1.9%	0.3%	8.1%	2.2%	0.6%
Antipsychotics	17.6%	21.3%	2.0%	19.2%	19.6%	3.2%
Anxiolytics	9.6%	29.5%	7.8%	14.9%	43.4%	13.6%
Antidepressants	24.1%	62.2%	10.6%	40.4%	64.9%	20.9%
Any psychiatry visit	24.7%	39.4%	7.9%	5.7%	9.3%	1.7%
Psychiatric diagnosis	21.3%	100.0%	0.0%	20.7%	100.0%	0.0%
