# Supplementary material for: Disparities in Utilization of Psychiatry Services Among Home Care Clients: The Tale of Two Canadian Jurisdictions
Source: Front Psychiatry. 2021 Sep 17;12:712112. doi: 10.3389/fpsyt.2021.712112 (PMC8484907; doi:10.3389/fpsyt.2021.712112)
Supplement: Supplementary file 1 [file Table_1.DOCX]

Supplementary Material: Mental Health Diagnoses

1. RAI-Home Care Assessment: Any psychiatric diagnosis diagnosed by a physician that is present and affects the client status, regardless of whether home care is monitoring or treating it.
2. ICD-9 from physician visit records:

| ICD9 3-digit code | diagnosis |
| --- | --- |
| 291 | Alcohol-induced mental disorders |
| 292 | Drug-induced mental disorders |
| 294 | Persistent mental disorders due to conditions classified elsewhere |
| 295 | Schizophrenic disorders |
| 296 | Episodic mood disorders |
| 297 | Delusional disorders |
| 298 | Other nonorganic psychoses |
| 300 | Anxiety, dissociative and somatoform disorders |
| 301 | Personality disorders |
| 302 | Sexual and gender identity disorders |
| 303 | Alcohol dependence syndrome |
| 304 | Drug dependence |
| 305 | Nondependent abuse of drugs |
| 306 | Physiological malfunction arising from mental factors |
| 307 | Special symptoms or syndromes, not elsewhere classified |
| 308 | Acute reaction to stress |
| 309 | Adjustment reaction |
| 310 | Specific nonpsychotic mental disorders due to brain damage |

1. ICD-10 from inpatient hospital discharge records:

| ICD10 3-digit code | diagnosis |
| --- | --- |
| F06 | Other mental disorders due to brain damage and dysfunction and to physical disease |
| F07 | Personality and behavioural disorders due to brain disease, damage and dysfunction |
| F09 | Unspecified organic or symptomatic mental disorder |
| F10-F19 | Mental and behavioural disorders due to psychoactive substance use |
| F20-F29 | Schizophrenia, schizotypal and delusional disorders |
| F30-F39 | Mood [affective] disorders |
| F40-F48 | Neurotic, stress-related and somatoform disorders |
| F50-F59 | Behavioural syndromes associated with physiological disturbances and physical factors |
| F60-F69 | Disorders of adult personality and behaviour |
| F80-F89 | Disorders of psychological development |
| F99 | Unspecified mental disorder |

Selected Mental Health Diagnoses Groups

| Diagnoses | ICD9 (physician billing records) | ICD10 (any hospitalization) |
| --- | --- | --- |
| Anxiety | 300 | F40, F41, F42 |
| Psychoses | 295, 296, 297, 298, 299 | F20 to F29, F30, F31, F34 |
| Acute reaction to stress or adjustment disorder | 308, 309 | F43 |
| Special symptoms including sleep or pain disorders | 307 |  |
| Alcohol or drug related | 303, 304 | F10 to F19 |
| Personality disorder | 301 | F60, F61, F62, F69 |

Informed by: Statistics Canada. Table A: ICD-9 and ICD-10 codes for selected causes of death [Internet]. Ottawa, ON; 2015 Jul. Report No.: 82-003–X. Available from: <https://www150.statcan.gc.ca/n1/pub/82-003-x/2009004/article/11034/tables/tbla-eng.htm>
